# Supplementary material for: Premature vision drives aberrant development of response properties in primary visual cortex
Source: bioRxiv. 2025 Jun 2:2025.03.13.643139. Originally published 2025 Mar 13. Preprint. [Version 2] doi: 10.1101/2025.03.13.643139 (PMC11952534; doi:10.1101/2025.03.13.643139)
Supplement: Supplement 1 [file NIHPP2025.03.13.643139v2-supplement-1.pdf]

bioRxiv preprint doi: https://doi.org/10.1101/2025.03.13.643139; this version posted June 2, 2025. The copyright holder for this preprint (which was not certified by peer review) is the author/funder, who has granted bioRxiv a license to display the preprint in perpetuity. It is made available under aCC-BY 4.0 International license.

Figure 3 – figure supplement 1. Premature eye opening alters L50 and H50 values. A) Temporal frequency L50 values across the experimental conditions. E01contra cells exhibited significantly lower L50 values values than control animals ($p<0.002$, LMEM). **B)** EO2 cells, by contrast, exhibited widened bandwidths by showing increased H50 values ($p<0.004$, LMEM). **C)** Considering the absolute value of the temporal frequency response (where deviations above or below background firing rates contributed to response), EO1contra cells again exhibited significantly lower L50 values compared to control cells ($p<0.00029$, LMEM). EO1ipsi cells exhibited a slight increase in L50 values ($p<0.0148$, LMEM). **D)** EO1ipsi cells showed a substantial increase in absolute H50 cut off values ($p<0.00169$, LMEM).

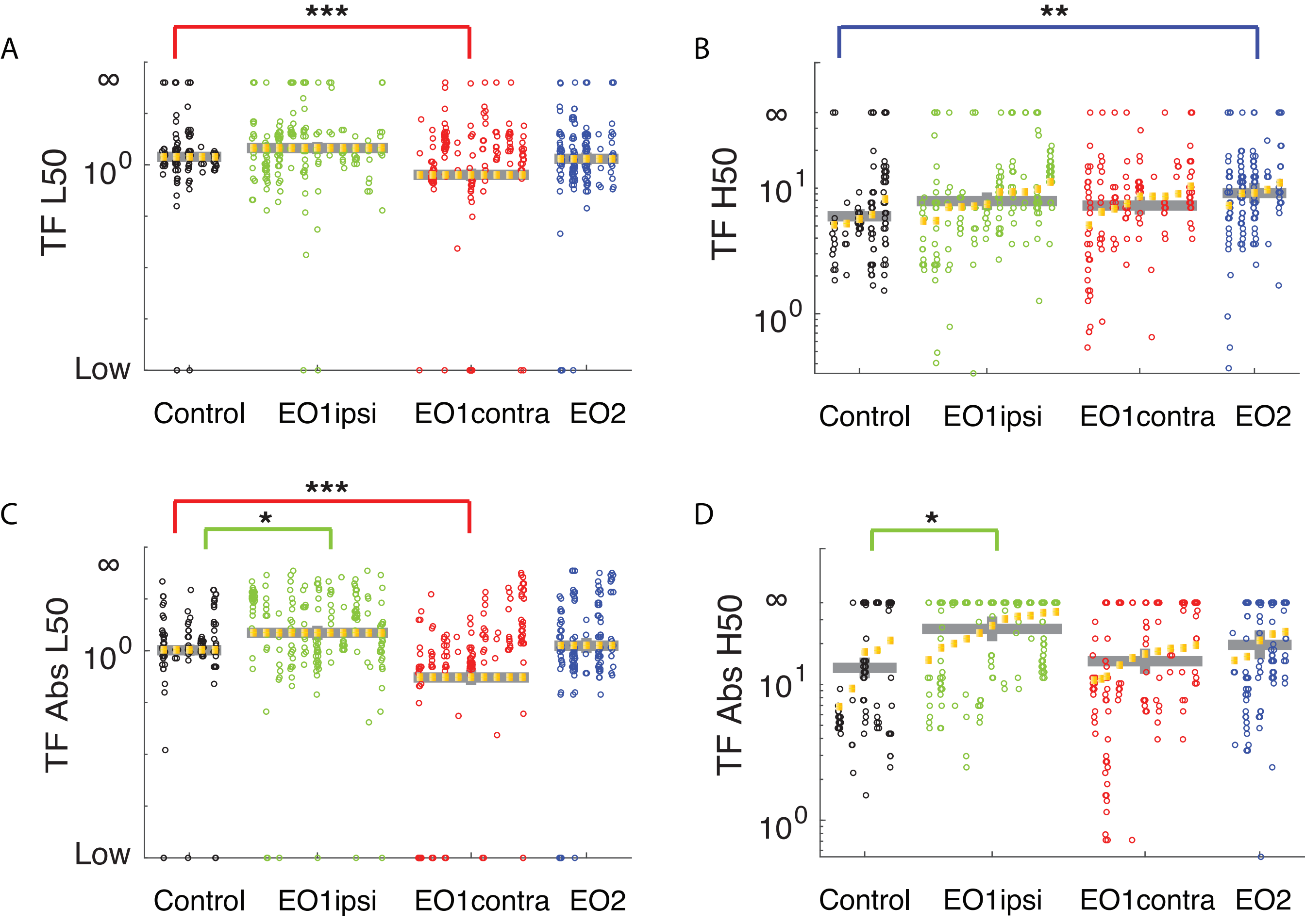
A
***
TF L50
∞
10^0
Low
Control
EO1ipsi
EO1contra
EO2
B
**
TF H50
∞
10^1
10^0
Control
EO1ipsi
EO1contra
EO2
C
***
*
TF Abs L50
∞
10^0
Low
Control
EO1ipsi
EO1contra
EO2
D
*
TF Abs H50
∞
10^1
10^0
Control
EO1ipsi
EO1contra
EO2
